# The Poor Man's Home.—XXV

**Published:** 1898-02-12

**Authors:** 


### MODERN SOCIOLOGY.

### THE POOR MAN'S HOME.—XXV.

### MODEL LODGING-HOUSES (*concluded*).

BESIDES the houses we have spoken of, there is in Glasgow one lodging-house of a superior kind. In the "Orient" Home the regular charge is 6d. per night, or, if the beds are taken by the week, 3s. per week. For this sum the lodger gets a cubicle entirely to himself, without anyone above or below him, a good hair mattress, and two clean sheets every night. There is also a locker in the bedroom where he can keep his clothes. If they desire it, two friends can join their resources, and have a moderate-sized room with two beds in it, which has also the advantage of a wash-stand and looking-glass within its bounds. In all the lodging-houses the beds are aired daily, and though lacking in decoration, the places are kept thoroughly clean, perhaps more so than many more pretentious hotels. A small army of scrubbers is kept steadily at work, and each house is under the charge of a resident superintendent. The proprietor also pays frequent visits without notice, both by day and by night. The superintendent gets the profit of the store attached to each lodging-house, as well as a fixed salary; but the articles sold are inspected frequently by the proprietor, and the prices to be charged are fixed by him. While everything possible is done for the comfort of the inmates of the homes, Mr. Burns is very strict about them being kept decent and orderly. No alcoholic liquors are permitted to be introduced, and swearing or rioting, or anything calculated to disturb the peace of the inmates is liable to cause the offender to be summarily dismissed. That these institutions are appreciated by those whom they are intended to benefit may be inferred from the fact that 80 per cent. of the inmates are permanent lodgers. Many of them are known to Mr. Burns personally, and are ready to bring to him the story of all their affairs. While the majority are casual labourers, there is a substantial minority who by no means belong to the poorest class, but who for lack of relations stay in these houses, and are glad of the companionship they afford.

These Glasgow experiments have attained so much notoriety that it was necessary to speak of them first and at some length. All model lodging-houses of recent date are modelled more or less upon their example; but the house erected by the London County Council in Parker Street, Drury Lane, claims to have improved upon the Glasgow model. In the Glasgow cubicles there is the danger that the currents of air may pass over the compartments without changing the air within them, and that, therefore, the same air may be breathed over and over again throughout the night by the occupant. To avoid this, in the London building the dormitories are arranged in two large halls in three tiers of galleries. The cubicles are ranged round these galleries, and each cubicle has a window, by which a current of fresh air is admitted at a height of 4½ ft. from the floor. The air space allowed each sleeper is 435 cubic feet. The sides of each cubicle reach the ceiling of the gallery; but at the other end, where the door is, the height is only 5½ ft., so that the current of air may circulate into the central well, from which the foul air is drawn by a ventilator in the roof. To prevent the occupants of the cubicles from feeling any ill effects from this draught, the feet of the sleepers are set towards the window. In each hall there is a box for a night watchman, placed so as to command the entire dormitory accommodation. The cubicles are arranged for one person only, and have a lock. The other arrangements are in most respects similar to those of Glasgow; but in Parker Street there is a glass-covered yard for exercise, and here all the conveniences for day use are situated. Here are found the closets, lavatories, washing and drying rooms, &c. On each floor, however, there is a closet for use during the night. The charge per night is 5d., and at this rate the enterprise is expected to return 3 per cent. on the cost, besides providing a sufficient sinking-fund to liquidate the original cost of the building.

Even better than the Parker Street house, however, is Rowton House, Vauxhall. This was erected by Lord Rowton, at a cost of about £30,000, and is, according to the testimony of an American observer, "a magnificent structure, admirably fitted throughout, and in all salient respects a residential club. There is adequate lavatory and bathing accommodation, while the sanitary conveniences are such as would do credit to a first-class hotel." A special feature is that lodgers have the choice of cooking their own meals or having them served at fixed rates. In Rowton House there are 470 cubicles, the price of each being 6d. per night. Each is provided with a chair and an iron bedstead with a spring mattress, a hair mattress, bolster, blankets, sheets, and coverlet—in short, the complete furnishing of any bed. The price charged for this is 6d. per night. The first year showed a profit of 5 per cent., and the enterprise has now been taken over by a company, of which Lord Rowton is chairman. During the last three months before the company entered into possession the profits averaged 6 per cent. The work is being extended, and similar lodging-houses built in different parts of London. In a recent number of the *Nineteenth Century*, the question was asked, "Why not a Rowton House for Clerks?" Why not, indeed? Many clerks earn less than an artisan, as little as a casual labourer. They can ill afford to pay more than 3s. a week for their lodging, and for this sum the ordinary landlady gives them but poor accommodation. It can hardly be doubted that the lodgings of young men of the poorer middle class are inferior in both comfort and healthfulness to the houses we have been describing. Why should all the efforts of philanthropists be concentrated on those who wear rough clothes and who do only manual labour? In a complex civilisation like ours, the "horny-handed son of toil" is not the only worker, not even the hardest worker; and the very fact that the clerk has to spend more out of earnings, which are no greater than those of the working man (so called *par excellence*), on clothes, on railway journeys, and the like, makes cheap good lodgings and cheap good food a greater boon to him than to the other. It may be said that the clerk is free to go to Rowton House even now. He is, but, except in rare instances, he won't do it. Like draws to like, and the sense of incompatibility of temper and taste, which would keep him from free association with those around him, would soon drive him away.

